# Supplementary figures and images for: Chicken cGAS Senses Fowlpox Virus Infection and Regulates Macrophage Effector Functions
Source: Front Immunol. 2021 Feb 1;11:613079. doi: 10.3389/fimmu.2020.613079 (PMC7901977; doi:10.3389/fimmu.2020.613079)

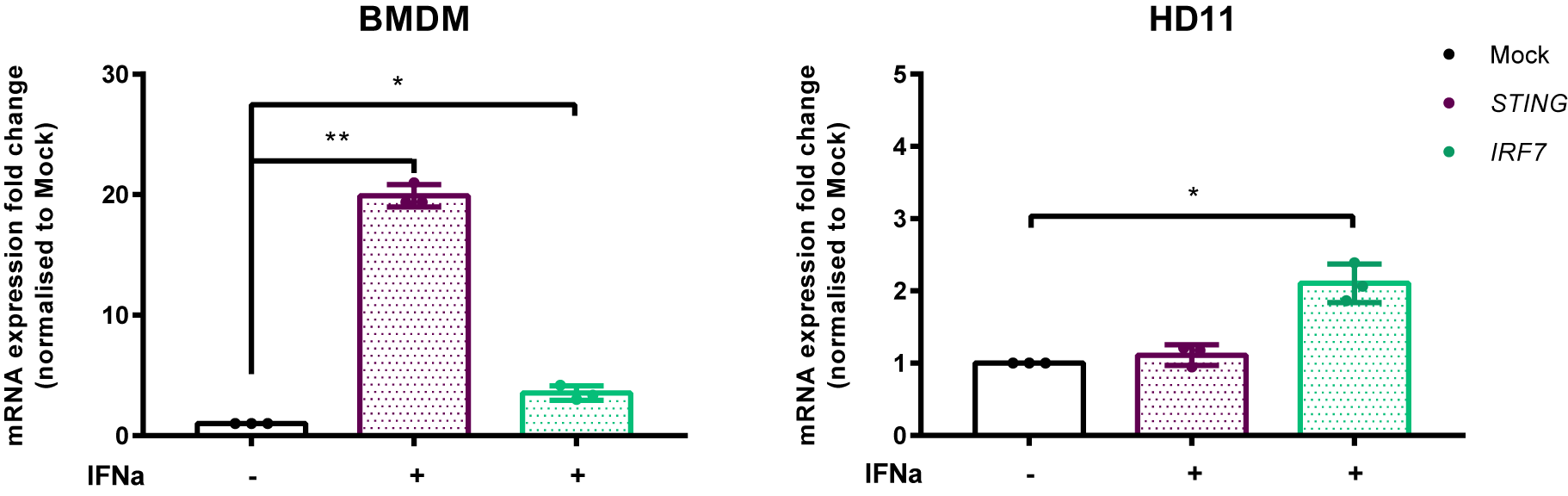

Supplement: Supplementary Figure1 — Effect of IFNα priming on expression levels of STING and IRF7 in BMDM and HD11. BMDM or HD11 cells were treated with IFNα for 6 h and transcription of STING and IRF7 measured by qRT-PCR 6 h later. Data is representative of two or more replicates. [file Image_1.tif]

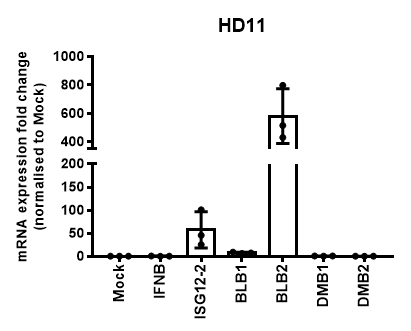

Supplement: Supplementary Figure 2 — BLB2 but not BLB1 is an ISG in HD11 cells. HD11 cells were treated with IFNα for 6 h and the indicated genes were measured by qRT-PCR. Data is representative of two or more replicates. [file Image_2.png]

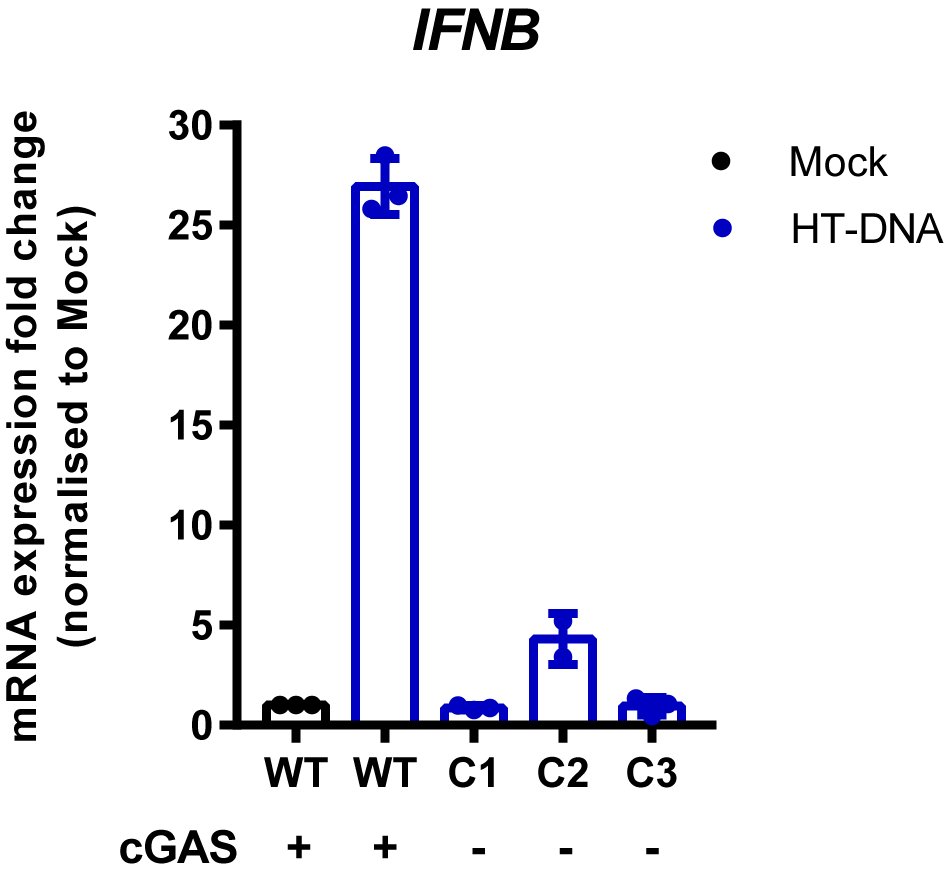

Supplement: Supplementary Figure 3 — cGAS is essential for intracellular DNA-dependent IFN-I transcription in HD11 cells. WT or three individual cGAS knockout clones with different indels were stimulated with HT-DNA (2 μg/ml) and IFNB transcription measured by qRT-PCR 6 h later. Data is representative of two or more replicates. [file Image_3.tif]

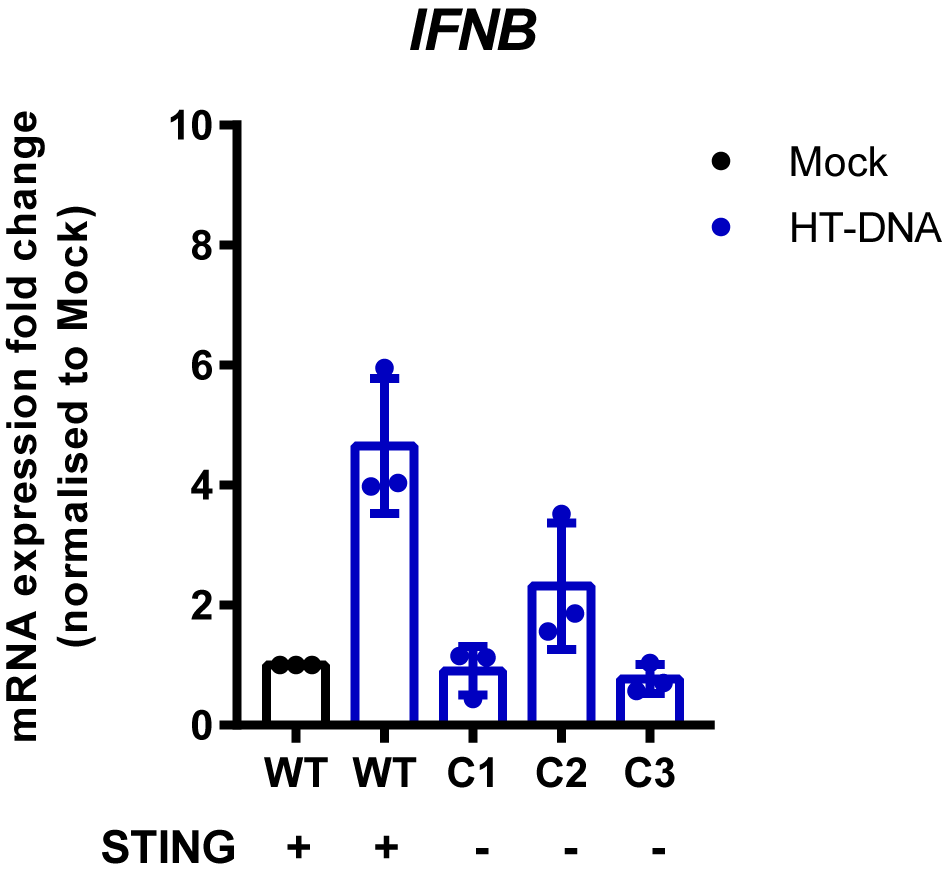

Supplement: Supplementary Figure 4 — STING is essential for intracellular DNA-dependent IFN-I transcription in HD11 cells. WT or three individual cGAS knockout clones with different indels were stimulated with HT-DNA (2 μg/ml) and IFNB transcription measured by qRT-PCR 6 h later. Data is representative of two or more replicates. [file Image_4.tif]

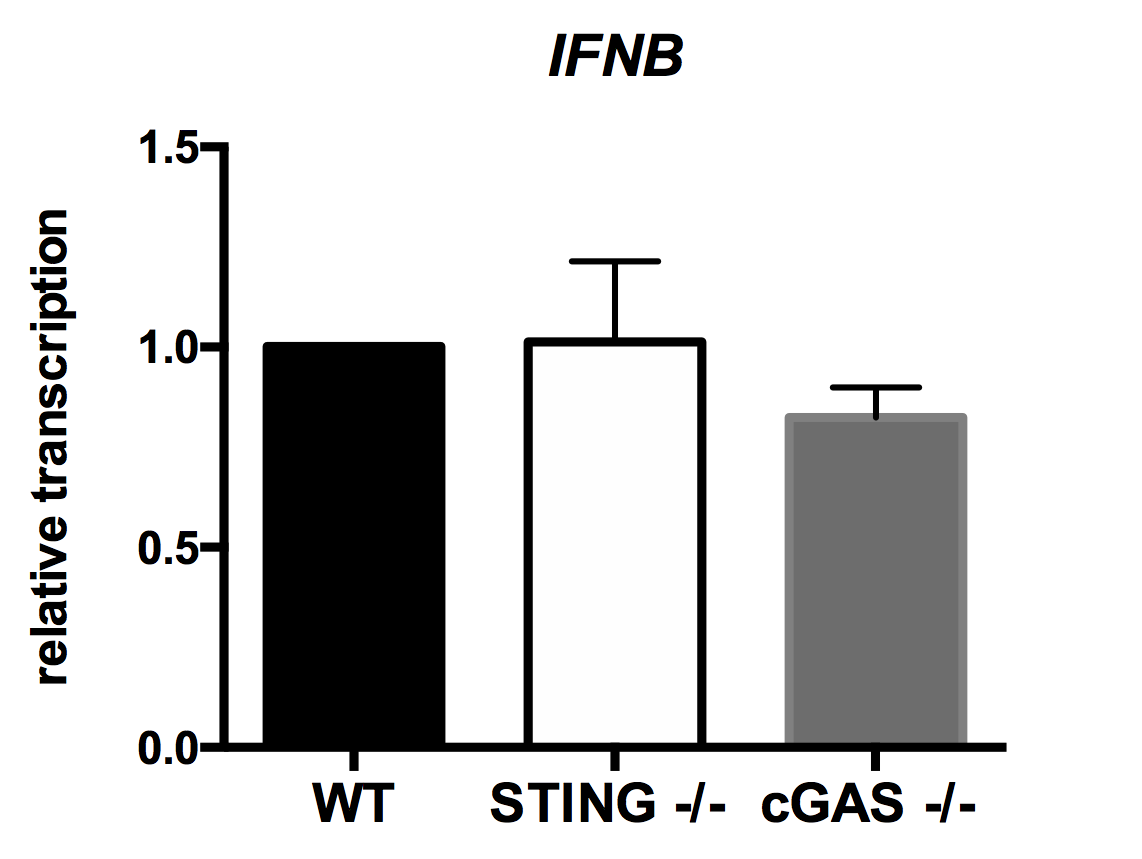

Supplement: Supplementary Figure 5 — STING or cGAS loss does not significantly alter tonic IFN-I transcription. IFNB transcription measured by qRT-PCR in WT, cGAS KO or STING KO HD11 cells (data shown relative to WT cells). [file Image_5.png]
